# Efficacy and Safety of Combination Therapy With Immune Checkpoint Inhibitors and Chemotherapy With Gemcitabine and Nab‐Paclitaxel in Pancreatic Cancer: A Systematic Review

**DOI:** 10.1002/cam4.71637

**Published:** 2026-02-17

**Authors:** Hanye Sohrabi, Seyed Mohammad Ghavam, Sina Azadnajafabad, Nima Rezaei

**Affiliations:** ^1^ School of Medicine Tehran University of Medical Sciences Tehran Iran; ^2^ Non‐Communicable Diseases Research Center, Endocrinology and Metabolism Population Sciences Institute Tehran University of Medical Sciences Tehran Iran; ^3^ Research Center for Immunodeficiencies (RCID), children's Medical Center Tehran University of Medical Sciences Tehran Iran; ^4^ Network of Immunity in Infection, Malignancy and Autoimmunity (NIIMA) Universal Scientific Education and Research Network (USERN) Tehran Iran; ^5^ Department of Immunology, School of Medicine Tehran University of Medical Sciences Tehran Iran

**Keywords:** chemotherapy, CTLA‐4 inhibitors, immune checkpoint inhibitors, pancreatic neoplasms, PD‐L1 inhibitors, systematic review

## Abstract

**Background:**

Pancreatic cancer (PC) is a lethal malignancy with a 5‐year survival rate of 13% as the 7th leading cause of cancer‐related death worldwide. Most patients are ineligible for resection at diagnosis. Monotherapy with immune checkpoint inhibitors (ICIs) has shown limited success in PC, with objective response rates below 5% and median overall survival rarely exceeding 6 months. This systematic review evaluates the efficacy and safety of combining gemcitabine and nab‐paclitaxel with ICIs in pancreatic cancer.

**Objective:**

To assess the efficacy and safety of combining gemcitabine and nab‐paclitaxel chemotherapy with ICIs in patients with pancreatic cancer.

**Methods:**

A comprehensive search of PubMed, Scopus, and Web of Science was performed using predefined keywords. Eligible studies included original research articles employing interventional, cohort, case–control, or cross‐sectional designs. Case reports, case series, and review articles were excluded. Data extraction and quality assessment using the Mixed Methods Appraisal Tool (MMAT, 2018) were conducted independently by two reviewers.

**Results:**

Of 1904 search results, seven studies met inclusion criteria: four clinical trials and three retrospective cohorts. Sample sizes ranged from 17 to 180 participants with advanced or locally advanced PC. Median overall survival was ~15 months (range: 9.8–16.7) versus 8–9 months for chemotherapy alone. Progression‐free survival ranged from 5.5–9 versus 3.5–5.5 months. Objective response rates varied between 18%–50% for combination therapy and 23%–29% for chemotherapy. Grade 3–4 adverse events were mainly hematologic (anemia, neutropenia) and neurologic (fatigue, peripheral neuropathy). ICIs included PD‐1 inhibitors (pembrolizumab, nivolumab, toripalimab, camrelizumab, tislelizumab, sintilimab), PD‐L1 inhibitor (durvalumab), and CTLA‐4 inhibitor (tremelimumab).

**Conclusion:**

Combining ICIs with gemcitabine and nab‐paclitaxel appears feasible and safe, with signals of improved efficacy compared with chemotherapy alone. However, evidence remains limited, and further large‐scale trials are warranted to confirm survival benefits and optimize therapeutic strategies in pancreatic cancer.

## 1 Introduction

Pancreatic cancer (PC) is currently the third leading cause of cancer‐related death in the United States and the seventh in the world. With a five‐year relative survival rate of only 13%, it has the lowest survival rate among all malignant neoplasms. In 2025, an estimated 67,440 new cases of PC and 51,980 related deaths are expected to occur [1, 2]. The most common pathological subtype of PC is Pancreatic Ductal Adenocarcinoma (PDAC), accounting for 90% of cases [3]. The aggressive nature of PC is largely due to its early tendency to metastasize. By the time of diagnosis, most PCs have already spread, and only 10%–15% of cases are categorized as localized [4].

Pancreatic cancer has a multifactorial etiology. Among modifiable lifestyle‐related risk factors, smoking exhibits the strongest association with pancreatic adenocarcinoma [5]. Additionally, excessive alcohol consumption (> 30 g per day), chronic pancreatitis, obesity, and diets rich in processed meats, high‐fructose beverages, and saturated fats are linked to an increased risk of pancreatic cancer [6]. Genetic predisposition also plays a critical role, with variants that impair the DNA damage repair system contributing to disease susceptibility. The most prevalent of these are BRCA2, BRCA1 (associated with hereditary breast and ovarian cancer syndrome), and ATM (linked to ataxia‐telangiectasia syndrome) [7]. Less common but clinically significant variants in mismatch repair (MMR) genes, including MLH1, MSH2, MSH6, and PMS2, are also observed in pancreatic adenocarcinoma and are characteristic of Lynch syndrome [8].

The diagnosis of pancreatic cancer relies on imaging modalities in cases of clinical suspicion. CT, MRI, endoscopic ultrasound (EUS) with fine‐needle aspiration (FNA), and PET scans are valuable diagnostic tools for detecting pancreatic masses. Imaging also plays a crucial role in assessing metastatic disease. However, the gold standard for diagnosis remains histopathological evaluation [9]. The role of laboratory findings in pancreatic cancer diagnosis is limited and not fully reliable. The most commonly associated biomarker, CA 19–9, lacks sufficient sensitivity and specificity to serve as a dependable screening tool [10]. However, it can be useful for prognostication, as well as monitoring disease remission or recurrence [11].

The latest National Comprehensive Cancer Network (NCCN) guidelines recommend systemic chemotherapy for all stages of PC. The preferred regimens are either FOLFIRINOX (made up of folinic acid, fluorouracil, irinotecan, and oxaliplatin) or Gemcitabine plus albumin‐bound paclitaxel (Nab‐Paclitaxel) [11]. However, the overall survival rates achieved with standard chemotherapy regimens for pancreatic cancer remain low and unsatisfactory, with median survival ranging from approximately 8.5 months with gemcitabine plus nab‐paclitaxel to 11.1 months with the FOLFIRINOX regimen [12, 13, 14].

Immune checkpoints are regulatory proteins on the surface of immune cells, such as T‐cells, that interact with other cell‐surface proteins to modulate immune responses. The most clinically relevant immune checkpoint proteins are cytotoxic T‐lymphocyte antigen‐4 (CTLA‐4) and programmed cell death‐1 (PD‐1) [15]. These proteins are expressed on T‐cells after activation and serve to downregulate T‐cell activity [16, 17]. Tumor cells exploit this mechanism to evade immune surveillance by altering immune system responses, with CTLA‐4 and PD‐1 playing pivotal roles in this process [18].

Immune checkpoint inhibitors (ICIs) are a class of immunotherapy drugs that have shown promising results in various cancers, including non‐small cell lung cancer (NSCLC), advanced melanoma, Hodgkin's lymphoma, renal cell carcinoma (RCC), bladder cancer, and head and neck squamous cell carcinoma (HNSCC) [19]. However, the tumor microenvironment in PC is considered immunologically “cold,” characterized by limited T‐cell infiltration due to a dense stromal barrier, a higher prevalence of regulatory T‐cells (T‐regs) and suppressor macrophages, and the secretion of inhibitory cytokines such as IL‐10, TGF‐β, and chemokine receptor 5 [20, 21, 22]. These factors contribute to immune evasion in PC.

Studies such as those by Royal et al. [23], Brahmer et al. [24], and Sharma et al. [25] have demonstrated that ICI monotherapy yields limited efficacy in PDAC. Notably, Marabelle et al. [26] reported promising results for microsatellite instability‐high (MSI‐high) PDACs, though these represent only about 1% of all PDAC cases. Additionally, a study by O'Reilly et al. [27] indicated that even dual ICI therapy does not achieve the desired outcomes in PDAC treatment.

While chemotherapy is traditionally known to suppress the immune system, recent evidence suggests that it can also enhance antitumor immune responses. This effect is believed to result from increased immunogenicity and antigen presentation following tumor cell apoptosis induced by chemotherapy [28]. Plate et al. [29] reported this effect in PC patients treated with gemcitabine. The synergistic effect of combining ICIs with chemotherapy has been demonstrated along with favorable safety profiles in previous systematic reviews of other cancer types [30, 31]. These findings warrant further investigation of this combination therapy in PC, a particularly lethal malignancy.

Despite the growing interest in chemoimmunotherapy, there remains a lack of comprehensive synthesis of evidence regarding the efficacy and safety of combining ICIs with gemcitabine and Nab‐Paclitaxel in PC treatment. To address this gap, we conducted a systematic review to evaluate the clinical outcomes and safety concerns associated with this combination therapy. The findings from this review are anticipated to provide valuable insights for clinicians aiming to improve prognosis and survival rates in pancreatic cancer patients.

## 2 Method

This systematic review was conducted in accordance with the Preferred Reporting Items for Systematic Reviews and Meta‐Analyses (PRISMA) guidelines [32]. This review aimed to systematically analyze all studies up to September 17, 2024, involving pathology‐confirmed pancreatic cancer (PC) patients treated with a combination regimen of Gemcitabine and Nab‐Paclitaxel chemotherapy alongside immune checkpoint inhibitors.

### 2.1 Search Strategy and Data Sources

A comprehensive systematic search was conducted in PubMed, Web of Science (WOS), and Scopus using relevant Medical Subject Headings (MeSH) terms for pancreatic cancer and immunotherapy on September 17, 2024. The complete list of keywords is provided in Table S1.

### 2.2 Eligibility Criteria

We included English‐language observational and interventional studies on adult patients (≥ 18 years) with advanced or locally advanced pancreatic cancer who were ineligible for surgery. Eligible studies investigated the combination of Gemcitabine and Nab‐Paclitaxel chemotherapy with immune checkpoint inhibitors as first‐line treatment. Studies were excluded if they used chemotherapy regimens other than gemcitabine plus nab‐paclitaxel, if they did not evaluate this combination as a first‐line therapy, or if they contained incomplete or insufficient data. We also excluded studies that were not published in English, as well as review articles, case reports, case series, and cross‐sectional studies.

### 2.3 Data Extraction

Two independent reviewers extracted data from the included studies, with discrepancies resolved by a third reviewer. Extracted data included: first author's name, year of publication, country of origin, clinical trial identifier, study type, sample size, participants' age and sex, prior therapy, intervention model, masking, randomization, inclusion criteria, immune checkpoint inhibitor type, study arms, outcomes including objective response rate (ORR) which is the proportion of patients whose tumor shrinks or disappears after treatment, disease control rate (DCR) which is the percentage of patients whose disease does not progress including stable disease and partial or complete responses, overall survival (OS) which is the length of time from diagnosis or treatment start until death from any cause, 1‐year overall survival (1‐Year OS) which is the percentage of patients who are alive 1 year after diagnosis or the start of treatment, progression free survival (PFS) which refers to the duration during which a patient remains alive without disease progression, and grade 3 and 4 adverse events (AEs) which are Severe (Grade 3) or life‐threatening (Grade 4) side effects from treatment.

### 2.4 Risk of Bias (ROB) and Quality Assessment

The quality of included studies was assessed using the Mixed Methods Appraisal Tool (MMAT) version 2018, which evaluates different study designs within systematic reviews. This tool assesses qualitative research, randomized controlled trials, non‐randomized studies, quantitative descriptive studies, and mixed‐methods studies based on criteria such as study objectives, population characteristics, randomization, blinding, data completeness, and confounding factors. The MMAT comprises two universal screening questions (S1–S2) applicable to all study designs (S1: Are there clear research questions? S2: Do the collected data allow addressing the research questions?), followed by five design‐specific categories (1: Qualitative Studies, 2: Quantitative Randomized Controlled Trials, 3: Quantitative non‐randomized studies, 4: Quantitative descriptive studies, 5: Mixed methods studies) that evaluate methodological adequacy according to 5 design‐specific questions (criteria). Each criterion is rated as Yes [1], No (0), or can't tell based on the information reported. Consistent with MMAT guidance, we did not compute an overall score; instead, we present the full pattern of criteria ratings to illustrate methodological strengths and limitations [33].

## 3 Results

### 3.1 Study Selection

**FIGURE 1:**
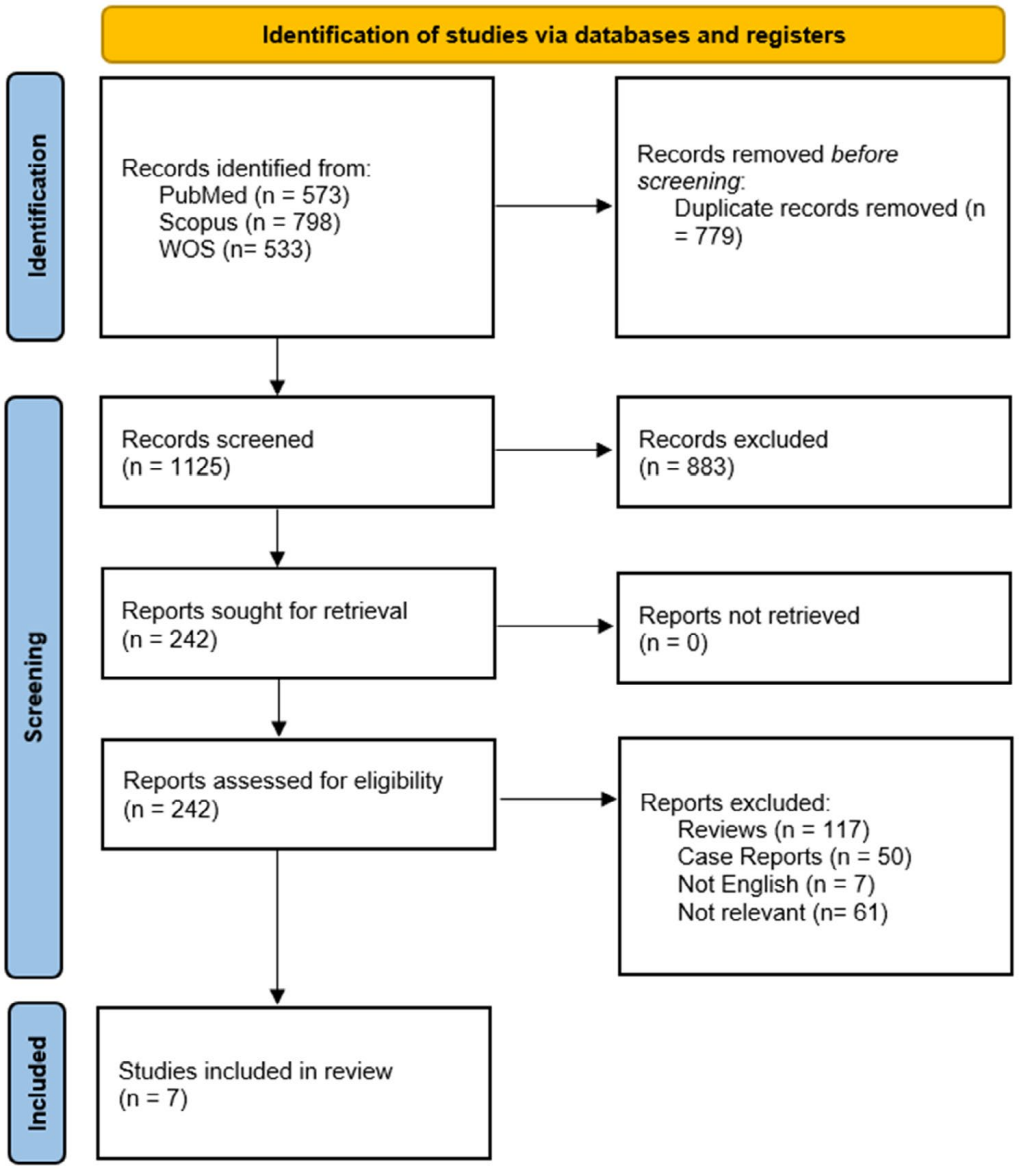
Study selection flow chart.

From the 1904 studies identified through searches in PubMed, Scopus, and Web of Science, 1125 articles remained after manual removal of duplicates for title and abstract screening. Following the initial screening, 20 articles were selected for full‐text review, of which 7 studies met the eligibility criteria (Figure 1) [34, 35, 36, 37, 38, 39, 40].

### 3.2 Characteristics of the Included Studies

Across the 7 studies, sample sizes ranged from 17 to 180 patients (median = 48). All participants were patients with advanced pancreatic cancer, with 6 studies focusing specifically on pancreatic adenocarcinoma [34, 35, 37, 38, 39, 40], while Cheng et al. [36] included additional pancreatic cancer subtypes. Geographically, three studies were conducted in China [35, 36, 40], three in the United States [37, 38, 39], and one in Canada [34]. Study participants had an Eastern Cooperative Oncology Group (ECOG) performance status of 0, 1, or 2 or a Karnofsky performance status (KPS) above 80% before enrollment. Except for Weiss et al. [39], all studies included treatment‐naïve patients. These criteria were employed to evaluate patients before study enrollment, as detailed in Table 1.

**TABLE 1:** Characteristics of the included studies.

First author	Year	Country	Trial ID	Study type	Sample size	Median age	Female (%)	Prior therapy	Intervention model	Randomization/masking	Main inclusion criteria	ICI type	Study arms		Key outcomes
													Arm A	Arm B	
Weiss	2018	USA	NCT02331251	Phase Ib/phase II trial	17	56 (46–66)	11 (64.7%)	5 (29.4%)	single arm (only arm 3 is reported)	open‐label	17 proven mPDAC, KPS > = 70%	PD‐1 inhibitor (Pembrolizumab)	NA	NA	RP2D
				Phase II trial	11			0 (0.0%)	single arm		Chemotherapy‐naïve mPDAC	PD‐1 inhibitor (Pembrolizumab)			DCR, OS
Wainberg	2020	USA	NCT02309177	Part 1‐phase I trial	50	67.5 (43–86)	22 (44%)	Arm A‐ part 1 received prior chemotherapy	2‐part, 2‐arm	open‐label	Chemotherapy‐naïve locally advanced or metastatic PDAC	PD‐1 inhibitor (Nivolumab)	Nivolumab + nab‐paclitaxel	Nivolumab + nab‐paclitaxel + gemcitabine	RP2D, AEs
				Part 2‐ phase I trial				0							OS, PFS, AEs
Zhang	2022	China	NA	Retrospective Cohort	48	> 60 years: 17 (35.4%)	22 (45.8%)	16 (51.6%)	2‐arm	NR	Advanced mPDAC (stage 4)	PD‐1 inhibitor (one of: Toripalimab, Camrelizumab, Tislelizumab, Sintilimab)	Gemcitabine+ nab‐paclitaxel	Gemcitabine + nab‐paclitaxel + anti‐PD‐1	OS, PFS, ORR, DCR, HR, AEs
Renouf	2022	Canada	NCT02879318	Phase II trial	180	64 (29–81)	87 (48.33%)	0	2‐arm	Randomized	mPDAC	PD‐L1 inhibitor (Durvalumab), CTLA‐4 inhibitor (Tremelimumab)	Gemcitabine +nab‐paclitaxel	Gemcitabine+ nab‐paclitaxel + durvalumab +tremelimumab	OS, PFS, ORR
Padron	2022	USA	NCT03214250	Phase Ib/II trial	34+ 421 (historical control)	62.5 (47–75)	14 (41%)	0	3 arms (1 arm included) + 1 historical control	Open‐label, Randomized	mPDAC	anti PD‐1 (Nivolumab)	Gemcitabine +nab‐paclitaxel	Gemcitabine+ nab‐paclitaxel+ Nivolumab	RP2D, 1‐year OS, ORR, DCR, PFS, DOR
Chen	2023	China	NA	Retrospective Cohort	27	64 (46–77)	9 (33.33%)	0	Single arm	NA	Locally advanced or mPDAC	PD‐1 inhibitor (one of: Pembrolizumab, Nivolumab, Toripalimab, Sintilimab, Tislelizumab, Camrelizumab)	NA	NA	AEs, OS, PFS, ORR
Cheng	2023	China	NA	Retrospective Cohort	53	67 (45–79)	23 (43.4%)	0	2‐arm	NA	Stage 3/4 pancreatic cancer	PD‐1 inhibitor (one of: Pembrolizumab, Sintilimab, Camrelizumab, Tislelizumab)	Gemcitabine +nab‐paclitaxel	Gemcitabine+ nab‐paclitaxel + PD‐1 inhibitor	PFS, OS, AEs

Abbreviations: 1‐Year OS, 1‐year overall survival; AEs, adverse events; AG, gemcitabine‐based chemotherapy regimen; DCR, disease control rate; DOR, duration of response; HR, hazard ratio; ICI, immune checkpoint inhibitors; NA, not applicable; NR, not reported; ORR, objective response rate; OS, overall survival; PFS, progression free survival; RP2D, recommended Phase 2 dose.

All studies used intravenous Gemcitabine (1000 mg/m^2^) and intravenous Nab‐Paclitaxel (125 mg/m^2^). In four studies, this combination was administered on days 1 and 8 of a 21‐day cycle, with immune checkpoint inhibitors (ICI) administered on day 1 [35, 36, 39, 40], whereas in three studies, treatment followed a 28‐day cycle, with chemotherapy on days 1, 8, and 15, and ICI on days 1 and 15 [34, 37, 38]. The majority of ICI regimens included anti‐PD‐1/PD‐L1 inhibitors [35, 36, 37, 38, 39, 40], except for Renouf et al. [34], who additionally utilized a CTLA‐4 inhibitor (Tremelimumab). The specific drugs used are detailed in Table 2.

**TABLE 2:** Therapeutic regimens.

Treatment		Dosing	Timing
Chemotherapy	Gemcitabine	1000 mg/m2	days 1,8, [and 15]
	Nab‐Paclitaxel	125 mg/m2	days 1,8, [and 15]
PD‐1 inhibitors	Pembrolizumab	2 mg/kg or 200 mg	day 1 [and 15]
	Nivolumab	3 mg/kg or 240 mg	day 1 [and 15]
	Toripalimab	240 mg	day 1 [and 15]
	Camrelizumab	200 mg	day 1 [and 15]
	Tislelizumab	200 mg	day 1 [and 15]
	Sintilimab	200 mg	day 1 [and 15]
	Durvalumab	1500 mg	day 1 [and 15]
CTLA‐4 inhibitor	Tremelimumab	75 mg	day 1

### 3.3 Quality and Risk of Bias Assessment

As previously mentioned, study quality and risk of bias were assessed using the Mixed Methods Appraisal Tool (MMAT) [33]. The quality assessment results for the 7 included studies are presented in Table 3. All non‐randomized studies received acceptable scores in terms of participant generalizability, adequacy of intervention and outcome measurements, completeness of reported results, and consideration of confounding factors (e.g., baseline patient characteristics and expected interventions) [35, 36, 38, 39, 40]. The two randomized studies scored well in terms of comparability between groups, completeness of outcome data, and proper intervention administration; however, they did not score for blinding due to their open‐label design [34, 37].

**TABLE 3:** Quality assessment of included studies using the mixed methods appraisal tool (MMAT 2018).

Studies	Criteria from the Mixed Methods Appraisal Tool (MMAT)																										
	S1	S2	1.1	1.2	1.3	1.4	1.5	2.1	2.2	2.3	2.4	2.5	3.1	3.2	3.3	3.4	3.5	4.1	4.2	4.3	4.4	4.5	5.1	5.2	5.3	5.4	5.5
Weiss et al., 2018	1	1											1	1	1	1	1										
Wainberg et al., 2020	1	1											1	1	1	1	1										
Zhang et al.,2022	1	1											1	1	1	1	1										
Renouf et al., 2022	1	1						0	1	1	0	1															
Padron et al., 2022	1	1						1	0	1	0	1															
Chen et al., 2023	1	1											1	1	1	1	1										
Cheng et al., 2023	1	1											1	1	1	1	1										

*Note:* Each study was evaluated according to the MMAT 2018 checklist, which includes two universal screening questions (S1: Are there clear research questions?; S2: Do the collected data allow addressing the research questions?) and five design‐specific categories: (1) Qualitative studies, (2) Quantitative randomized trials, (3) Quantitative non‐randomized studies, (4) Quantitative descriptive studies, and (5) Mixed‐methods studies. Each category comprises five methodological criteria assessing, respectively, the appropriateness of design, adequacy of data collection, validity of analysis, interpretation of results, and coherence or integration of findings. Items were rated as 1 = Yes, 0 = No, or blank = Can't tell.

### 3.4 Survival Outcomes

#### 3.4.1 Overall Survival (OS)

Since all studies followed patients until death, overall survival (OS) was reported across all trials(Table 4). The median overall survival observed in the studies included was 15 months. In Wainberg et al. [38] and Renouf et al. [34], the OS for patients receiving combination chemotherapy + immunotherapy was 9.9 months and 9.8 months, respectively. However, in Renouf et al. [34], this did not differ significantly from the 8.8‐month OS of the chemotherapy‐only group. Weiss et al. [39] reported an OS of 15 months for combination therapy. In Zhang et al. [40], OS improved significantly from 9.3 months (chemotherapy monotherapy) to 12.1 months (combination therapy). Padron et al. [37] reported a one‐year survival rate of 57.7%, which was significantly higher than the 35% historical control. The OS in this study was 16.7 months, significantly higher than the 8.5 months in the historical control group. Chen et al. [35] reported an OS of 16.4 months, while in Cheng et al. [36], OS improved from 8 months (chemotherapy alone) to 15 months (combination therapy).

#### 3.4.2 Progression‐Free Survival (PFS)

Across the reviewed studies, progression‐free survival (PFS) ranged from 5.5 to 8 months (Table 4). In Wainberg et al. [38] and Renouf et al. [34], PFS reached 5.5 months, showing no statistically significant advantage for combination therapy over chemotherapy alone. Similarly, while Zhang et al. [40] demonstrated a significant OS improvement, PFS did not significantly differ between groups. However, in Weiss et al. [39], PFS was reported at 9 months, and Chen et al. [35] reported 6.4 months. In Cheng et al. [36], PFS improved significantly from 3.5 months (chemotherapy alone) to 8 months (combination therapy) (Table 4).

**TABLE 4:** Reported outcomes of the included studies.

Author		Weiss	Wainberg	Zhang	Renouf	Padron	Chen	Cheng
Patient Status prior to therapy		Chemotherapy‐naïve KPS ≥ 80%	Locally advanced or metastatic PDAC ECOG performance status 0 or 1	Advanced metastatic (stage 4) ECOG score of ≤ 2 points	Metastatic PDAC ECOG performance status 0 or 1	Metastatic PDAC ECOG performance status 0 or 1	Unresectable locally advanced or metastatic PDAC ECOG performance status 0 or 1	Unresectable stage III and IV pancreatic cancer
Prior Therapy		7 from 17 patients	No prior therapy	No previous treatment or at least 6 months past the previous treatment	No previous treament for metastatic disease	No prior therapy unless more than 4 months before enrollment	No prior therapy	No prior therapy
ORR	AG group	NA	NA	25.8% (9.5%–42.1%)	23%	29 (25–34)	NA	NA
	AG + ICI group	27%	18% (8.6–31.4)	35% (10–60.6)	30.30%	50% (32–68)	37.04%	NA
	*p*‐value	NA	NA	Not significant (> 0.05)	Not significant (*p*‐value = 0.28)	NA	NA	NA
DCR (PR + SD)	AG group	NA	NA	74.2% (57.9–90.5)	57.40%	48% (43–53)	NA	NA
	AG + ICI group	100%	64% (49.2–77.1)	82.4% (62.1–100)	70.60%	74% (56–87)	74.08%	NA
	*p*‐value	NA	NA	Not significant (> 0.05)	Not significant (*p*‐value = 0.09)	NA	NA	NA
OS/1‐year OS	AG group	NA	NA	9.3 (8.8–9.8)	8.8	35% median OS = 8.5	NA	8
	AG + ICI group	15	9.9 (6.74–12.16)	12.1 (8.1–16.1)	9.8	57.7% median OS = 16.7 (9.8–18.4)	66.7% 16.4 (14.21–18.6)	15
	*p*‐value	NA	NA	Significant (*p*‐value < 0.001)	Not Significant (*p*‐value = 0.72)	Significant (*p*‐value = 0.006)	NA	Significant (*p*‐value < 0.001)
PFS	AG group	NA	NA	4.9 (4.1–5.7)	5.4	5.5	NA	3.5
	AG + ICI group	9.1	5.5 (3.25–7.2)	5 (3.3–6.7)	5.5	6.4 (5.2–8.8)	6.4 (3.98–8.75)	8
	*p*‐value	NA	NA	Not Significant (*p* = 0.154)	Not Significant (*p*‐value = 0.91)	Not interpretable	NA	Significant (*p*‐value = 0.002)
Grade 3/4 Events	AG group	NA	NA	38.7% (20.5%–56.9%)	76%	4%	NA	50%
	AG + ICI group	71%	96%	35.3% (10%–60.6%)	84%	≥ 20%	2 (6.4%)	55.60%
Conclusion		GNP can be safely given to chemotherapy naïve PDAC patients. Efficacy appears to be slightly improved over previously reported results for standard weekly × 3 every 28 day gemcitabine and nab‐paclitaxel dosing. CNI change may be prognostic for OS.	Overall, the clinical results of this study do not support further investigation. Efficacy beyond the historical control of nab‐paclitaxel plus gemcitabine was not observed.	Reduced Risk of death by approximately 80% in AG + ICI group (HR = 0.203, *p*‐value < 0.001)	1‐ these data indicate that the combination of gemcitabine, nab‐paclitaxel and dual ICI does not offer significant clinical benefit over chemotherapy alone in an unselected population of patients with mPDAC 2‐ KRAS wildtype (in 10%–15% of mPDAC) is associated with better OS and PFS and is considered a prognostic marker	the combination of nivolumab and gemcitabine plus nab‐paclitaxel for PC resulted in a higher 1‐year OS rate than gemcitabine plus nab‐paclitaxel alone (57.7% vs. 35%) in a phase II trial	hepatic metastasis, treatment cycles (less than 6 cycles), and ECOG performance status [1] were significantly associated with less OS. Treatment cycles (less than 6 cycles) and ECOG PS (= 1) were significantly associated with less PFS. CA 19–9 significantly decreases with chemo+ICI, however is not significantly associated with OS and PFS	PD‐1 blockade combined with nab‐paclitaxel plus gemcitabine demonstrated superior efficacy to chemotherapy alone for unresectable stage III/IV PC patients

Abbreviations: 1‐Year OS, 1‐year overall survival; AEs, adverse events; PFS, progression free survival; AG, gemcitabine‐based chemotherapy regimen; DCR, disease control rate; DOR, duration of response; HR, hazard ratio; ICI, immune checkpoint inhibitors; NA, not applicable; NR, not reported; ORR, objective response rate; OS, overall survival; RP2D, recommended phase 2 dose. The darker gray shade shows those cells presenting significant results.

### 3.5 Safety‐Related Outcomes and Adverse Events

The pooled adverse events (AEs) (grade ≥ 3) are presented in Table 5. Across seven included studies involving a total of 257 patients, 184 grade 3–4 AEs were identified. Hematologic AEs were the most frequently reported category, accounting for 85 events (46.2% of all AEs). Among non‐hematologic AEs, neurologic events were the most prevalent, comprising 53 events (28.8%).

**TABLE 5:** Safety related outcomes and grade 3,4 adverse events.

Sample Size		Weiss, 2018	Wainberg, 2020	Zhang, 2022 (chemo + ICI arm)	Renouf, 2022 (chemo+ ICI arm)	Padron, 2022	Chen, 2023	Cheng, 2023 (chemo + ICI arm)
		15	2	50	17	119	27	27
Grade 3/4 AEs		11 (73%)	1 (50%)	49 (98%)	6 (35%)	100 (84%)	2 (7%)	15 (55%)
Hematologic	Leukopenia	0 (0%)	0 (0%)	6 (12%)	6 (35%)	0 (0%)	1 (3%)	6 (22%)
	Anemia	0 (0%)	0 (0%)	18 (38%)	3 (17%)	0 (0%)	1 (3%)	3 (11%)
	Neutrophilia	0 (0%)	0 (0%)	0 (0%)	0 (0%)	0 (0%)	0 (0%)	0 (0%)
	Neutropenia	7 (46%)	0 (0%)	10 (20%)	6 (35%)	0 (0%)	0 (0%)	5 (18%)
	Thrombocytopenia	3 (20%)	0 (0%)	3 (6%)	0 (0%)	0 (0%)	0 (0%)	3 (11%)
	Febrile Neutropenia	0 (0%)	0 (0%)	0 (0%)	0 (0%)	7 (6%)	0 (0%)	0 (0%)
Hepatobiliary	Hepatic Dysfunction	0 (0%)	0 (0%)	0 (0%)	0 (0%)	0 (0%)	0 (0%)	2 (7%)
	Bile Duct Stenosis	0 (0%)	0 (0%)	0 (0%)	0 (0%)	7 (6%)	0 (0%)	0 (0%)
	Biliary Tract Infections	0 (0%)	0 (0%)	0 (0%)	0 (0%)	11 (9%)	0 (0%)	0 (0%)
	Transaminase Elevation	2 (13%)	0 (0%)	5 (10%)	6 (35%)	0 (0%)	0 (0%)	0 (0%)
	ALP Elevation	0 (0%)	1 (50%)	0 (0%)	0 (0%)	0 (0%)	0 (0%)	0 (0%)
Respiratory	Immune‐related Pneumonia	0 (0%)	0 (0%)	0 (0%)	0 (0%)	0 (0%)	0 (0%)	2 (7%)
	Lung Infections	0 (0%)	0 (0%)	0 (0%)	0 (0%)	6 (5%)	0 (0%)	0 (0%)
	Dyspnea	0 (0%)	0 (0%)	0 (0%)	0 (0%)	8 (7%)	0 (0%)	0 (0%)
	Hypoxia	0 (0%)	0 (0%)	3 (6%)	0 (0%)	0 (0%)	0 (0%)	0 (0%)
Cardiovascular	Thromboembolic Events	0 (0%)	0 (0%)	0 (0%)	0 (0%)	16 (15%)	0 (0%)	0 (0%)
	Limb Edema	0 (0%)	0 (0%)	0 (0%)	0 (0%)	5 (4%)	0 (0%)	0 (0%)
	Hypertension	0 (0%)	0 (0%)	0 (0%)	0 (0%)	3 (3%)	0 (0%)	0 (0%)
Neurologic	Fatigue	0 (0%)	0 (0%)	4 (8%)	0 (0%)	24 (20%)	0 (0%)	0 (0%)
	Peripheral Sensory Neuropathy	0 (0%)	0 (0%)	8 (16%)	0 (0%)	13 (11%)	0 (0%)	0 (0%)
	Generalized Muscle Weakness	0 (0%)	0 (0%)	3 (6%)	0 (0%)	1 (1%)	0 (0%)	0 (0%)
Gastrointestinal	Immune‐related Enteritis	0 (0%)	0 (0%)		0 (0%)	0 (0%)	0 (0%)	1 (3%)
	Diarrhea	0 (0%)	0 (0%)	5 (10%)	0 (0%)	6 (5%)	0 (0%)	0 (0%)
	Abdominal Pain	0 (0%)	0 (0%)	5 (10%)	0 (0%)	6 (5%)	0 (0%)	0 (0%)
	Vomiting	0 (0%)	0 (0%)	3 (6%)	0 (0%)	7 (6%)	0 (0%)	0 (0%)
	Nausea	0 (0%)	0 (0%)	5 (10%)	0 (0%)	0 (0%)	0 (0%)	0 (0%)
Systemic	Sepsis	0 (0%)	0 (0%)	0 (0%)	0 (0%)	13 (11%)	0 (0%)	0 (0%)
Nephrologic	Renal Calculi	0 (0%)	0 (0%)	0 (0%)	0 (0%)	2 (2%)	0 (0%)	0 (0%)
Cutaneous	Maculopapular Rash	0 (0%)	0 (0%)	0 (0%)	0 (0%)	7 (6%)	0 (0%)	0 (0%)
	Reactive Cutaneous Capillary Hyperplasia	0 (0%)	0 (0%)	0 (0%)	2 (11%)	0 (0%)	0 (0%)	0 (0%)
Water, Electrolyte, and Sugar	Hypokalemia	0 (0%)	0 (0%)	7 (14%)	0 (0%)	0 (0%)	0 (0%)	0 (0%)
	Hyperglycemia	0 (0%)	0 (0%)	5 (10%)	0 (0%)	0 (0%)	0 (0%)	0 (0%)
	Hyponatremia	2 (13%)	0 (0%)	5 (10%)	0 (0%)	0 (0%)	0 (0%)	0 (0%)
	Dehydration	0 (0%)	0 (0%)	3 (6%)	0 (0%)	0 (0%)	0 (0%)	0 (0%)

## 4 Discussion

To the best of our knowledge, this systematic review is the first comprehensive analysis of the literature providing insights into the efficacy and safety of combination therapy with immune checkpoint inhibitors and chemotherapy with gemcitabine and nab‐paclitaxel in patients with nonresectable pancreatic cancer. The findings of the included studies suggest that this combination therapy is a relatively safe and effective approach for improving survival rates while minimizing serious adverse events in this patient population.

In terms of overall survival (OS), five out of seven studies reported an OS exceeding one year, which is generally not expected with chemotherapy alone [35, 36, 37, 39, 40]. Notably, four studies included control groups for direct comparison [34, 36, 37, 40], all of which demonstrated increased OS, with three reporting statistically significant improvements [36, 37, 40]. Progression‐free survival (PFS) ranged from 5.5 to 8 months, slightly exceeding the 5.5‐month PFS typically observed with gemcitabine and nab‐paclitaxel alone [41]. Four studies compared PFS between combination therapy and chemotherapy‐only control groups [34, 36, 38, 40], but only Cheng et al. [36] reported a significant increase in PFS.

All seven studies included in this review reported grade 3 or higher adverse events (AEs), with overall incidence rates ranging from 7.4% to 100%. Despite the occurrence of severe AEs, no treatment‐related deaths were documented, underscoring the safety of the combination therapy. Each study classified the combination regimen as safe within its specific context. The chemotherapy regimen of gemcitabine plus nab‐paclitaxel has been extensively studied, with its safety profile documented in multiple studies, including those by Zhang et al. [42] and Damm et al. [43]. Immunotherapy, including immune checkpoint inhibitors combined with chemotherapy, has also been evaluated in cancer treatment. A meta‐analysis reported a 68.3% overall incidence of grade 3 or higher AEs, with hematological toxicities being the most prevalent, particularly neutropenia, followed by anemia [44]. In our analysis, hematological AEs were the most commonly reported grade 3 or 4 toxicities, aligning with the findings of Zhang et al. [42], Damm et al. [43], and Zhou et al. [44]. However, while previous studies identified neutropenia as the most frequent hematological AE, our findings indicated anemia as the most common, with neutropenia being the second most prevalent. Regarding non‐hematological AEs, neurological complications were frequently observed, consistent with the results of Zhang et al. [42] and Damm et al. [43]. However, our analysis identified fatigue as the most commonly reported non‐hematological AE, whereas neuropathy was the predominant AE in those previous reviews.

Given the modest efficacy of chemotherapy alone in nonresectable pancreatic cancer, with OS typically under 1 year, the optimal treatment strategy remains uncertain [41]. The limited efficacy of chemotherapy may be addressed by incorporating immune checkpoint inhibitors, theoretically enhancing therapeutic outcomes [28]. Our study highlights the potential of this combination approach; however, additional randomized controlled trials with well‐defined control groups, adjusted for key factors such as age and sex, are needed to refine our understanding of its efficacy and safety.

Moreover, further advances in pancreatic cancer (PC) management will likely rely on emerging diagnostic and therapeutic technologies. Artificial intelligence‐based approaches are increasingly being explored for early detection, risk stratification, and individualized treatment planning in gastrointestinal malignancies, including PC [45]. In addition, preclinical studies have identified novel biological and pharmacologic strategies that may enhance treatment responsiveness. For example, adiponectin has been shown to suppress pancreatic ductal adenocarcinoma (PDAC) progression [46], and combined modulation of paricalcitol and hydroxychloroquine has demonstrated potential to sensitize tumors to gemcitabine‐based therapy [47, 48]. Similarly, polymeric drug delivery systems and small‐molecule inhibitors are being investigated to improve drug solubility, tumor targeting, and therapeutic efficacy while overcoming chemoresistance [49, 50]. Moreover, inhibition of transcription factor YY1, an important mediator in inflammation‐related oncogenesis, may potentiate the effects of immune checkpoint blockade [51]. Together, these approaches represent promising avenues for future research aimed at improving early diagnosis, therapeutic precision, and survival outcomes in PDAC.

This systematic review is constrained by the limited number of studies investigating the combination of immune checkpoint inhibitors with gemcitabine and nab‐paclitaxel in pancreatic cancer. Only seven eligible trials were identified, of which four included control groups. However, the methods for adjusting baseline characteristics in these studies were not clearly defined, and one study used a historical control group, complicating direct comparisons. Additionally, heterogeneity in study designs, patient populations, treatment regimens, and outcome measures posed challenges in drawing definitive conclusions. Due to these limitations and the small number of studies, a meta‐analysis could not be conducted, restricting our ability to derive quantitative estimates of efficacy and safety. These factors highlight the need for more rigorous, well‐controlled clinical trials with standardized methodologies to better assess the therapeutic potential of this combination strategy.

The findings of this systematic review suggest that combining immune checkpoint inhibitors with gemcitabine and nab‐paclitaxel may enhance efficacy compared to chemotherapy alone in pancreatic cancer. Although data remain limited, available evidence indicates improved tumor response and potential survival benefits with this approach. Additionally, the safety profile appears acceptable, with adverse events consistent with those expected from chemotherapy and immunotherapy. However, further research is necessary to optimize toxicity management and identify the most suitable patient populations for this treatment strategy.

## Author Contributions

Hanye Sohrabi drafted the manuscript. Hanye Sohrabi and Seyed Mohammad Ghavam performed the study screening. Seyed Mohammad Ghavam, Sina Azadnajafabad, and Nima Rezaei contributed to revision and critical editing of the manuscript. Nima Rezaei supervised the study. All authors approved the final version of the manuscript.

## Funding

The authors have nothing to report.

## Ethics Statement

The authors have nothing to report.

## Conflicts of Interest

The authors declare no conflicts of interest.

## Supporting information


**Table S1:** Search strings and number of results.
**Table S2:** Eastern cooperative oncology group (ECOG) performance status.
**Table S3:** Karnofsky performance status (KPS).

## Data Availability

The authors have nothing to report.
