# Supplementary material for: Efficacy and Safety of Combination Therapy With Immune Checkpoint Inhibitors and Chemotherapy With Gemcitabine and Nab‐Paclitaxel in Pancreatic Cancer: A Systematic Review
Source: Cancer Med. 2026 Feb 17;15(2):e71637. doi: 10.1002/cam4.71637 (PMC12912933; doi:10.1002/cam4.71637)
Supplement: Supplementary file 1 — Table S1: Search strings and number of results. Table S2: Eastern cooperative oncology group (ECOG) performance status. Table S3: Karnofsky performance status (KPS). [file CAM4-15-e71637-s001.docx]

Table 1: Search strings and number of results

| Data Base | Search String | Number of Results |
| --- | --- | --- |
| PubMed | (((((((Pancreatic cancer*[Title/Abstract]) OR (Pancreatic neoplasm*[Title/Abstract])) OR (Pancreatic tumor*[Title/Abstract])) OR (Pancreatic malignancy[Title/Abstract])) OR (PDAC[Title/Abstract])) OR (Pancreatic ductal adenocarcinoma[Title/Abstract])) OR (Pancreatic neuroendocrine tumors[Title/Abstract])) AND (((((((((((((((((((((((((((((((Immune checkpoint inhibitor*[Title/Abstract]) OR (CTLA-4 inhibit*[Title/Abstract])) OR (CTLA4 inhibit*[Title/Abstract])) OR (T-lymphocyte-associated antigen 4 inhibit*[Title/Abstract])) OR (PD1 inhibit*[Title/Abstract])) OR (PD-1 inhibit*[Title/Abstract])) OR (programmed cell death-1 inhibit*[Title/Abstract])) OR (PD-L1 inhibit*[Title/Abstract])) OR (Programmed death-ligand 1 inhibit*[Title/Abstract])) OR (TIGIT inhibit*[Title/Abstract])) OR (VISTA inhibit*[Title/Abstract])) OR (PD-1H inhibit*[Title/Abstract])) OR (V-domain Ig suppressor of T cell activation inhibit*[Title/Abstract])) OR (Programmed death-1 homolog inhibit*[Title/Abstract])) OR (Ipilimumab[Title/Abstract])) OR (Tremelimumab[Title/Abstract])) OR (Nivolumab[Title/Abstract])) OR (Pembrolizumab[Title/Abstract])) OR (Atezolizumab[Title/Abstract])) OR (Avelumab[Title/Abstract])) OR (Durvalumab[Title/Abstract])) OR (Cemiplimab[Title/Abstract])) OR (Camrelizumab[Title/Abstract])) OR (Tislelizumab[Title/Abstract])) OR (Tiragolumab[Title/Abstract])) OR (Ociperlimab[Title/Abstract])) OR (Relatlimab[Title/Abstract])) OR (MBG453[Title/Abstract])) OR (TSR-022[Title/Abstract])) OR (Epacadostat[Title/Abstract])) OR (BMS-986205[Title/Abstract])) OR (Toripalimab[Title/Abstract])) OR (Sintilimab[Title/Abstract])) OR (Envafolimab[Title/Abstract])) OR (Socazolimab[Title/Abstract])) OR (Sotigalimab[Title/Abstract])) OR (KHK2455[Title/Abstract])) OR (Enoblituzumab[Title/Abstract])) OR (Ciforadenant[Title/Abstract])) | 573 |
| Scopus | ("Immune checkpoint inhibitor*" OR "CTLA-4 inhibit*" OR "CTLA4 inhibit*" OR "T-lymphocyte-associated antigen 4 inhibit*" OR "PD1 inhibit*" OR "PD-1 inhibit*" OR "programmed cell death-1 inhibit*" OR "PD-L1 inhibit*" OR "Programmed death-ligand 1 inhibit*" OR "TIGIT inhibit*" OR "VISTA inhibit*" OR "V-domain Ig suppressor of T cell activation inhibit*" OR "Programmed death-1 homolog inhibit*" OR "LAG-3 inhibit*" OR "Lymphocyte-activation gene 3 inhibit*" OR "TIM-3 inhibit*" OR "T-cell immunoglobulin and mucin-domain containing-3 inhibit*" OR "IDO1 inhibit*" OR "Indoleamine 2,3-dioxygenase inhibit*")  AND  "pancreatic cancer*" OR "Pancreatic neoplasm*" OR "Pancreatic tumor*" OR "Pancreatic malignancy" OR pdac OR "Pancreatic ductal adenocarcinoma" OR "Pancreatic neuroendocrine tumors" | 798 |
| Web of Science (WOS) | TS=("Immune checkpoint inhibitor*" OR "CTLA-4 inhibit*" OR "CTLA4 inhibit*" OR "T-lymphocyte-associated antigen 4 inhibit*" OR "PD1 inhibit*" OR "PD-1 inhibit*" OR "programmed cell death-1 inhibit*" OR "PD-L1 inhibit*" OR "Programmed death-ligand 1 inhibit*" OR "TIGIT inhibit*" OR "VISTA inhibit*" OR "V-domain Ig suppressor of T cell activation inhibit*" OR "Programmed death-1 homolog inhibit*" OR "LAG-3 inhibit*" OR "Lymphocyte-activation gene 3 inhibit*" OR "TIM-3 inhibit*" OR "T-cell immunoglobulin and mucin-domain containing-3 inhibit*" OR "IDO1 inhibit*" OR "Indoleamine 2,3-dioxygenase inhibit*") AND TS=("pancreatic cancer*" OR "Pancreatic neoplasm*" OR "Pancreatic tumor*" OR "Pancreatic malignancy" OR "PDAC" OR "Pancreatic ductal adenocarcinoma" OR "Pancreatic neuroendocrine tumors") | 533 |

Table 2: Eastern Cooperative Oncology Group (ECOG) performance status

| GRADE | ECOG PERFORMANCE STATUS |
| --- | --- |
| 0 | Fully active, able to carry on all pre-disease performance without restriction |
| 1 | Restricted in physically strenuous activity but ambulatory and able to carry out work of a light or sedentary nature, e.g., light house work, office work |
| 2 | Ambulatory and capable of all selfcare but unable to carry out any work activities; up and about more than 50% of waking hours |
| 3 | Capable of only limited selfcare; confined to bed or chair more than 50% of waking hours |
| 4 | Completely disabled; cannot carry on any selfcare; totally confined to bed or chair |
| 5 | Dead |

Table 3: Karnofsky Performance Status (KPS)

| Value | Level of Function Capacity | Definition |
| --- | --- | --- |
| 100 | Normal, no complaints, no evidence of disease | Able to carry on normal activity and to work; no special care needed |
| 90 | Able to carry on normal activity, minor signs or symptoms of disease |  |
| 80 | Normal activity with effort, some signs or symptoms of disease |  |
| 70 | Cares for self, unable to carry on normal activity or to do active work | Unable to work; able to live at home and care for most personal needs; various degrees of assistance needed |
| 60 | Requires occasional assistance but is able to care for most needs |  |
| 50 | Requires considerable assistance and frequent medical care |  |
| 40 | Disabled, requires special care and assistance | Unable to care for self; requires equivalent of institutional or hospital care; disease may be progressing rapidly |
| 30 | Severely disabled, hospitalization is indicated although death is not imminent |  |
| 20 | Hospitalization is necessary, very sick, active supportive treatment necessary |  |
| 10 | Moribund, fatal processes progressing rapidly |  |
| 0 | Dead |  |
